# Uptake and fate of surface modified silica nanoparticles in head and neck squamous cell carcinoma

**DOI:** 10.1186/1477-3155-9-32

**Published:** 2011-08-11

**Authors:** Emina Besic Gyenge, Xenia Darphin, Amina Wirth, Uwe Pieles, Heinrich Walt, Marius Bredell, Caroline Maake

**Affiliations:** 1 Institute of Anatomy, University of Zürich, Winterthurerstr. 190, 8057 Zürich, Switzerland; 2 University of Applied Sciences Northwestern Switzerland, School of Life Sciences, Institute of Chemistry and Bioanalytics, CH-4132 Muttenz, Switzerland; 3 Clinic for Cranio-Maxillofacial Surgery, University Hospital of Zürich, Frauenklinikstr. 24, 8091 Zürich, Switzerland

**Keywords:** nanoparticles, silica dioxide, surface properties, tumor cell line, uptake, endocytosis, cellular fate

## Abstract

**Background:**

Head and neck squamous cell carcinoma (HNSCC) is currently the eighth leading cause of cancer death worldwide. The often severe side effects, functional impairments and unfavorable cosmetic outcome of conventional therapies for HNSCC have prompted the quest for novel treatment strategies, including the evaluation of nanotechnology to improve e.g. drug delivery and cancer imaging. Although silica nanoparticles hold great promise for biomedical applications, they have not yet been investigated in the context of HNSCC. In the present in-vitro study we thus analyzed the cytotoxicity, uptake and intracellular fate of 200-300 nm core-shell silica nanoparticles encapsulating fluorescent dye tris(bipyridine)ruthenium(II) dichloride with hydroxyl-, aminopropyl- or PEGylated surface modifications (Ru@SiO_2_-OH, Ru@SiO_2_-NH_2_, Ru@SiO_2_-PEG) in the human HNSCC cell line UMB-SCC 745.

**Results:**

We found that at concentrations of 0.125 mg/ml, none of the nanoparticles used had a statistically significant effect on proliferation rates of UMB-SCC 745. Confocal and transmission electron microscopy showed an intracellular appearance of Ru@SiO_2_-OH and Ru@SiO_2_-NH_2 _within 30 min. They were internalized both as single nanoparticles (presumably via clathrin-coated pits) or in clusters and always localized to cytoplasmic membrane-bounded vesicles. Immunocytochemical co-localization studies indicated that only a fraction of these nanoparticles were transferred to early endosomes, while the majority accumulated in large organelles. Ru@SiO_2_-OH and Ru@SiO_2_-NH_2 _nanoparticles had never been observed to traffic to the lysosomal compartment and were rather propagated at cell division. Intracellular persistence of Ru@SiO_2_-OH and Ru@SiO_2_-NH_2 _was thus traceable over 5 cell passages, but did not result in apparent changes in cell morphology and vitality. In contrast to Ru@SiO_2_-OH and Ru@SiO_2_-NH_2 _uptake of Ru@SiO_2_-PEG was minimal even after 24 h.

**Conclusions:**

Our study is the first to provide evidence that silica-based nanoparticles may serve as useful tools for the development of novel treatment options in HNSCC. Their long intracellular persistence could be of advantage for e.g. chronic therapeutic modalities. However, their complex endocytotic pathways require further investigations.

## 1. Introduction

Head and neck squamous cell carcinoma (HNSCC) comprise a group of epithelial cancers that arise from e.g. the lips, the oral or nasal cavity, salivary glands, paranasal sinuses, pharynx or larynx [1]. With a worldwide incidence of more than 600'000 new cases per year, HNSCC accounts for about 6% of all malignant diseases diagnosed (http://globocan.iarc.fr). If detected early, patients have cure rates of about 90%. However, 60% of patients present with advanced disease or loco-regional lymph node metastasis at the time of diagnosis and have a poor prognosis [2,3].

Currently, treatment options for HNSCC patients include surgery, radiotherapy, chemotherapy or a combination of them [4,5]. Due to the distinct localization of these tumors in regions with anatomic structures important to e.g. breathing, mastication, swallowing or phonation, invasive treatment regimes frequently leading to severe functional impairments - often accompanied by unfavorable cosmetic outcomes. This is true despite significant advancements made in the reconstructive abilities over past two decades. Moreover, radiation may have long-term effects on surrounding healthy structures such as parts of the brain, the spinal cord or salivary glands. However, while surgery or radiation therapy is local, chemotherapy is applied systemically and may thus result in severe adverse effects e.g. on blood cell production (anaemia, neutropenia, thrombopenia), the mucosa (mucositis), the auditory and vestibular system (ototoxicity) or the kidneys (nephrotoxicity). Despite this aggressive therapeutic regime, to date many patients with advanced disease cannot be cured and more then half of them die within five years [6-8]. HNSCC is thus currently the eighth leading cause of cancer death worldwide.

To overcome at least some of the challenges in the therapy of patients with advanced HNSCC, the application of nanoparticles has been evaluated with regard to their advantages for chemotherapeutic/medicinal, radiation and imaging strategies. Previous data indicates that cytotoxic drugs such as mitoxantron, cisplatin or paclitaxel as well as the photosensitizer 5,10,15,20-tetrakis(meso-hydroxyphenyl)porphyrin (mTHPP) encapsulated in superparamagnetic, liposome, albumin or methoxy poly(ethylene glycol)-poly(lactide-co-glycolide) (MPEG-PLGA) nanoparticles or polymeric micelles not only exhibit potent antitumor activity, but also displayed reduced side effects [9-13]. Furthermore, it has been reported that beta-emitting radionuclides attached to liposomes showed promising results when applied intratumorally and gold nanoparticles or nanoparticles with antisense oligonucleotides against the gene ataxia-telangiectasia-mutated (ATM) improved radiosensitivity in rodent head and neck cancer models [14-16]. In addition, superior imaging in head and neck cancers resulted from the use of superparamagnetic iron oxide nanoparticles, gold nanoparticles or gadolinium-labelled phosphorescent polymeric nanomicelles [17-22].

In the past years, silica-based nanoparticles have gained increasing interest for medical applications because of their biocompatibility, versatility and stability. Numerous in-vitro and in-vivo studies pointed towards their great potential for improving the efficacy of therapeutic agents in tumor cells by e.g. circumventing solubility and stability problems of certain drugs or enabling targeted delivery and controlled release strategies [23-25]. Moreover, silica nanomaterials have been proposed as promising medical tools for biosensing [26,27] and imaging purposes [28].

However, to our knowledge, silica nanoparticles have not yet been investigated in the context of head and neck cancers. In this work, we assess the biological in-vitro behaviour of core-shell silica based nanoparticles on the HNSCC cell line UMB-SCC-745 with regard to their cytotoxicity, uptake, localization and intracellular fate.

## 2. Materials and methods

### 2.1. Synthesis of nanoparticles

Spherical core-shell silica nanoparticles encapsulating tris(bipyridine)ruthenium(II) dichloride [Ru(bpy)_3_]Cl_2 _as fluorescent dye were produced as described before [29]. The method is based on an oil-in-water microemulsion of n-hexanol-TritonX100-cyclohexane, [Ru(bpy)_3_]Cl_2_, tetraethyl-orthosilicate (TEOS) and ammonia. The surface chemistry of mono-shell silica nanoparticles was modified by the addition of a mixture of TEOS and other organosilanes, such as 3-aminopropyltriethoxysilane (APTES) to generate aminopropyl and hydroxyl functionalities (Ru@SiO_2_-NH_2 _and Ru@SiO_2_-OH) at the nanoparticle surface. Similarly, PEGylated [Ru(bpy)_3_]Cl_2_-labeled dual-shell nanoparticles (Ru@SiO_2_-PEG) have been synthesized as previously described, using a mixture of TEOS and bis(silylated)polyethylene glycol (SPEGS) for growth of a PEGylated second shell [30]. All the three types of nanoparticles have been fully characterized, as precedently described and have an average size ranging between 200 and 300 nm [30]. The surface charge and the hydrophilic character of nanoparticles have been explored based on their electrophoretic mobility in nanopure water at neutral pH (Zetasizer Nano ZS, Malvern Instruments Ltd., UK).

### 2.2. Cell Culture

The head and neck squamous carcinoma cell line UMB-SCC-745 was kindly provided by Dr. Robert Mandić, Department of Otolaryngology, Philips University, Marburg, Germany. The UMB-SCC-745 was derived from the tonsil tumor of a 48-year-old man and has a distinct p53 single point mutation and loss of heterozygosity [31].

#### Monolayer

UMB-SCC-745 cells were cultured under standard conditions (37°C, 5% CO_2_, 95% air atmosphere) in growth medium, i.e. RPMI Medium (Invitrogen, Basel, Switzerland) supplemented with 10% fetal calf serum (FCS, Sigma-Aldrich, Buchs, Switzerland), 1% HEPES (Invitrogen), 1% MEM non essential amino acids (Invitrogen) and 1% penicillin and streptomycin (Invitrogen). The growth medium was changed every second day. The passage of the cells was performed by trypsination (trypsin 1×, Invitrogen) when reaching confluence, in general every 2-3 days.

#### Multicellular spheroids (3D cell culture)

For generation of multicellular spheroids, we applied a modified hanging drop method [32]. Briefly, 96-well plates were coated with 60 μl of 1.5% agarose (Sigma-Aldrich) per well, in RPMI medium without FCS. Then 20 μl drops of UMB-SCC 745 cell solution (5000 cells/20 μl) were placed on the plate lid, the lid was positioned back to the plate and then kept overnight in the incubator (37°C, 5% CO_2_). The following day, 80 μl growth medium was added to the wells, the plates were shortly centrifuged and returned to the incubator. In order to avoid vibration, which has an influence on the formation of spheroids, the incubator should not be opened for the first 48 hours. After this initial time spheroids were stable in their form and reached the desired diameter of 150 μm two days later.

### 2.3. Proliferation assay

The cytotoxicity of nanoparticles was evaluated using a commercial cell proliferation assay (Cell Proliferation ELISA, BrdU, chemiluminescent, Roche, Basel, Switzerland). For this experiment the cells were cultured in black Greiner-96-well plates (2000 cells/well, Cellstar, Frickenhausen, Germany) with 100 μl growth medium at 37°C, 5% CO_2 _for 24 h. Subsequently the growth medium was replaced with fresh one containing Ru@SiO_2_-OH, Ru@SiO_2_-NH_2 _or Ru@SiO_2_-PEG nanoparticles at final concentrations ranging between 0.03 mg/ml - 0.5 mg/ml. Nanoparticles were ultrasonicated for 2 h before incubation to ensure their homogeneity. After nanoparticle incubation for 5 h, the cells were washed with phosphate buffered saline (PBS, Oxoid, Hampshire, United Kingdom) and incubated overnight with fresh growth medium containing BrdU-labeling agent. BrdU, which is incorporated only in viable cells during DNA synthesis, was detected with an ELISA immunoassay according to the recommendation of the manufacturer. The resulting signal was quantified by measuring the photons using a micro-plate luminometer with photomultiplier technology (BioTek, Luzern, Switzerland). The relative light units/second (rlu/s) directly correlates to the amount of DNA synthesis and hereby to the number of proliferating cells in the respective microcultures.

### 2.4. Exposure protocols of nanoparticles

For all experiments, nanoparticles were ultrasonicated for 2 h directly prior to use in cell culture.

For the uptake study the cells were seeded either on six-well plates (1'000'000 cells/well) for transmission electron microscopy (TEM) or on poly-L-lysine (PLL, 0.25 mg/ml, Sigma-Aldrich) -coated glass cover slips (50'000 cells, Hecht-Assistant, Sondheim, Germany) for confocal laser scanning microscopy (CLSM). The cells were then incubated with either Ru@SiO_2_-OH, Ru@SiO_2_-NH_2 _or Ru@SiO_2_-PEG nanoparticles (final concentrations 0.125 mg/ml) for different time periods (30 min, 1 h, 2 h, 5 h, 7 h, 12 h and 24 h) under cell culture conditions. After each time point cell aliquots were used for microscopic monitoring by CLSM and TEM.

Alternatively, multicellular spheroids were grown for 4 days in 96-well plates and also exposed to Ru@SiO_2_-OH and Ru@SiO_2_-NH_2 _nanoparticles for 5 h and 24 h, respectively, at final concentrations of 0.125 mg/ml under cell culture conditions. The nanoparticle distribution in spheroids was monitored only by CLSM.

For long-time experiments, cells were grown in six-well culture plates and incubated under cell culture conditions with Ru@SiO_2_-OH and Ru@SiO_2_-NH_2 _nanoparticles for 5 h (final concentrations 0.125 mg/ml). Following an extensive washing step with PBS, cells were directly passaged, re-seeded (500'000 cells/well) in cell culture plates and kept in culture until confluence (three days). The growth medium was exchanged every day. Passaging of the cells was continued until fifth passage. After each passage aliquots of the cells were used for evaluation by both CLSM and TEM.

For control experiments, cells or spheroids were cultured as above, but nanoparticle-containing medium was replaced by growth medium.

***Protocols for CLSM ***(TCS-SP2 and TCS-SP5, Leica, Heerbrugg, Switzerland)**: **After exposure to nanoparticles and washing steps, cells on cover slips were fixed for 15 min with PBS containing 1% paraformaldehyde (PFA, Sigma-Aldrich) and 0.33% saccharose (Sigma-Aldrich). Visualisation of nuclei were performed by incubation with 4'-6-diamidion-2-phenylindole (DAPI, 1 μg/ml, Roche) and mounted with GlycerGel mounting medium (Dako, Baar, Switzerland).

In experiments concerning multicellular spheroids, nuclei were stained with Hoechst staining dye (1 μg/ml, Sigma-Aldrich), which was added for the last hour of incubation. After incubation, the spheroids were collected, washed with PBS, fixed with PBS containing 1% PFA for 30 minutes, washed again with PBS and then monitored by confocal microscopy.

[Ru(byp)_3_]^2+ ^complexes were excited with a 458 nm laser and detected in the range of 570 - 650 nm. Visualisation of nuclei (DAPI and Hoechst staining) was achieved with an excitation wavelength of 350 nm and a detection wavelength range of 450 - 500 nm.

***Protocols for TEM ***(CM100, TEM, Philips, Guildford, UK)**: **After nanoparticle incubation and washing steps cells were fixed with 2.5% glutaraldehyde (GA, Electron Microscopy Sciences, Hatfield, USA) and 0.8% PFA in 0.05 M dimethylarsenic acid sodium salt trihydrate (Na-Caco, Merck, Darmstadt, Germany) buffer at 1:9 ratio for 30 minutes. The samples were washed once with 0.05 M Na-Caco buffer and then fixed for 1 h with 2% osmium-tetra-oxide and 3% potassium hexacyano-ferrate (II) trihydrate (Sigma-Aldrich) at 1:1 ratio. After washing and centrifugation, cell pellets were transferred to 2.5% bacto agar (Agar Scinetific, Wetzlar, Germany), dehydrated in 70-100% ethanol and embedded in embedding medium (Glycidether 100 (Promega); dodecenylsuccinic-anhydride (Sigma-Aldrich); nadic methyl anhydride (Sigma-Aldrich) and N, N-dimethylbenzylamin purum (Sigma-Aldrich) as activator) for 24 h at 80°C. Sections (70 nm) were contrasted with uranyl acetate dihydrate (Sigma-Aldrich) and lead (II) citrate (Sigma-Aldrich) for 20 minutes each.

### 2.5. Immunocytochemistry

UMB-SCC-745 cells cultured on PLL coated cover slides were incubated for 5 hours with Ru@SiO_2_-PEG, Ru@SiO_2_-OH and Ru@SiO_2_-NH_2 _nanoparticles at final concentrations of 0.125 mg/ml. After incubation cells were fixed for 15 min with 1% PFA in PBS, permeabilized with 0.01% Triton-X 100 (Roche) for 1.5 min, blocked for 30 min at room temperature with 0.1% bovine serum albumine (BSA, Calbiochem, San Diego, USA) and washed with PBS. For labelling of early endosomes, rabbit anti-EEA1 antibody (1:300, stock concentration 1.3 mg/ml, Sigma-Aldrich) was used. Rabbit anti-Rab7 antibody (1:300, stock concentration 1.2 mg/ml, Sigma-Aldrich) was used to visualize late endosomes and for labelling of Golgi apparatus mouse anti-GM130 antibody (1:500, stock concentration 0.7 mg/ml, Abcam, Cambridge, UK) was used. Cells were incubated with primary antibodies for 2 h at room temperature or overnight at 4°C, washed and incubated with FITC-labelled donkey anti-rabbit or anti-mouse antibodies, respectively (both 1:500, Sigma-Aldrich), together with DAPI (1 μg/ml) for 1 h at room temperature. Lysosomes and mitochondria were visualized with Lysotracker Red and Mitotracker Orange respectively (working concentration for both 300 nM, Invitrogen). For examination by CLSM (Leica), [Ru(byp)_3_]^2+ ^complexes and nuclei have been detected as described above, while for FITC excitation and detection wavelengths of 488 nm and 490-540 nm, respectively, have been used.

## 3. Results

**Figure 1:**
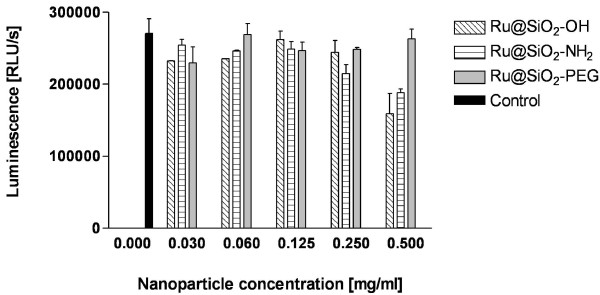
**Proliferation effects of different surface modified nanoparticles on UMB-SCC 745**. BrdU proliferation assays in UMB-SCC 745 cells after incubation (5 h) of nanoparticles with different surface modifications (Ru@SiO_2_-OH, Ru@SiO_2_-NH_2 _and Ru@SiO_2_-PEG) at concentration ranges of 0-0.5 mg/ml.

Electrophoretic mobility of Ru@SiO_2_-OH particles revealed a ζ-potential of -40 mV, which is in good agree, ζ-potentials of +11.3 mV and +4.29 mV have been obtained, respectively. As a prerequisite for our studies we first determined optimal concentrations of the different surface-modified nanoparticles in our in-vitro model (Figure 1). BrdU proliferation assays indicated for all types of nanoparticles that concentrations ranging between 0.03 - 0.125 mg/ml had no statisticment with the values measured for bare (non doped) SiO_2 _nanoparticles, whereas in the case of amino- and PEG-modified particlesally significant effect on cell proliferation compared to untreated controls. Ru@SiO_2_-PEG had no impact on cell growth even at higher concentrations (0.25 - 0.5 mg/ml). However, 0.25 and 0.5 mg/ml of Ru@SiO_2_-NH_2 _nanoparticles negatively affected proliferation rates, leading to an average of 21% and 31% reduced incorporation of BrdU, respectively. Ru@SiO_2_-OH nanoparticles diminished cell proliferation up to 41% at highest nanoparticle concentrations (0.5 mg/ml), while a reduction below 10% was observed at 0.25 mg/ml. Based on these results we decided to use concentrations of 0.125 mg/ml for all three Ru@SiO_2 _nanoparticles for further experiments.

**Figure 2:**
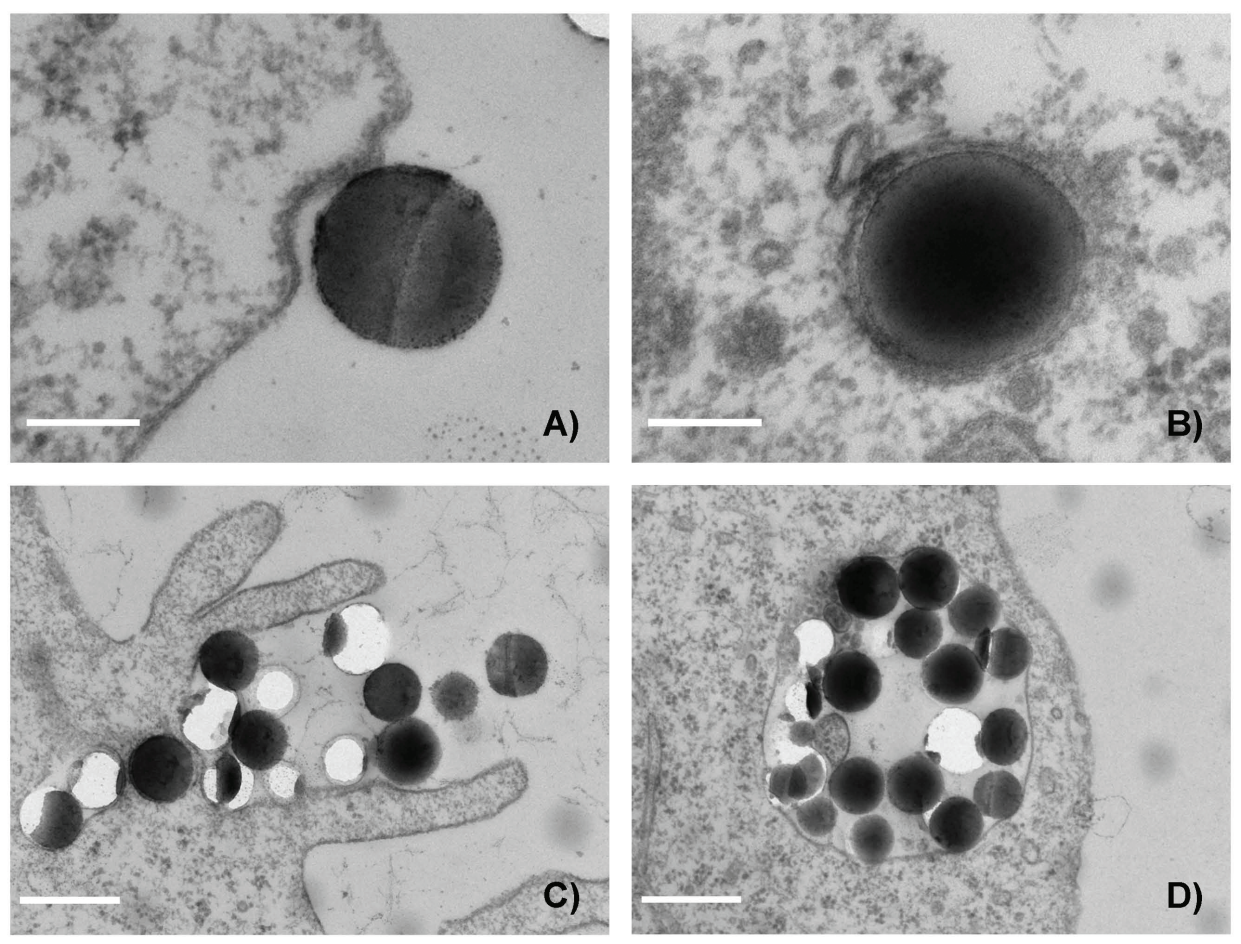
**Nanoparticle internalisation**. Transmission electron microscopy pictures of nanoparticle internalisation in UMB-SCC 745 exemplarily shown for Ru@SiO_2_-NH_2_. Uptake occurred either as single nanoparticle (A, B, scale bars = 100 nm), or nanoparticle clusters (C, D, scale bars = 500 nm).

**Figure 3:**
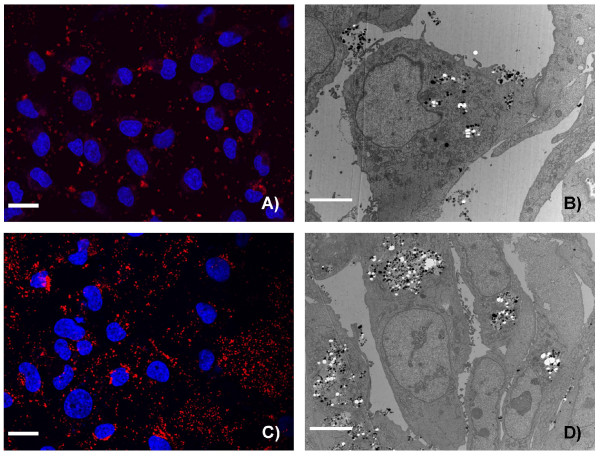
**Time dependent uptake of Ru@SiO_2_-OH nanoparticles**. Ru@SiO_2_-OH nanoparticle uptake over 2 h (A and B) and 24 h (C and D) in UMB- SCC 745. A, C: confocal laser scanning microscopy, showing nuclei in blue and Ru@SiO_2_-OH nanoparticles in red, scale bars = 20 μm. B, D: transmission electron microscopy, scale bars = 10 μm.

**Figure 4:**
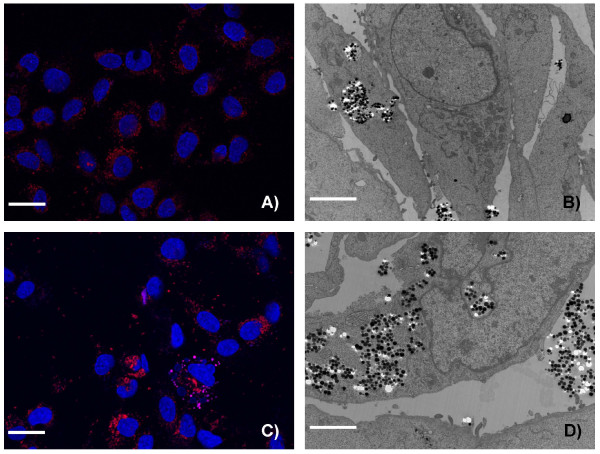
**Time dependent uptake of Ru@SiO_2_-NH_2 _nanoparticles**. Ru@SiO_2_-NH_2 _nanoparticles uptake over 2 h (A and B) and 24 h (C and D) in UMB-SCC 745. A, C: confocal laser scanning microscopy, showing nuclei in blue and Ru@SiO_2_-NH_2 _nanoparticles in red, scale bars = 20 μm. B, D: transmission electron microscopy, scale bars = 10 μm.

**Figure 5:**
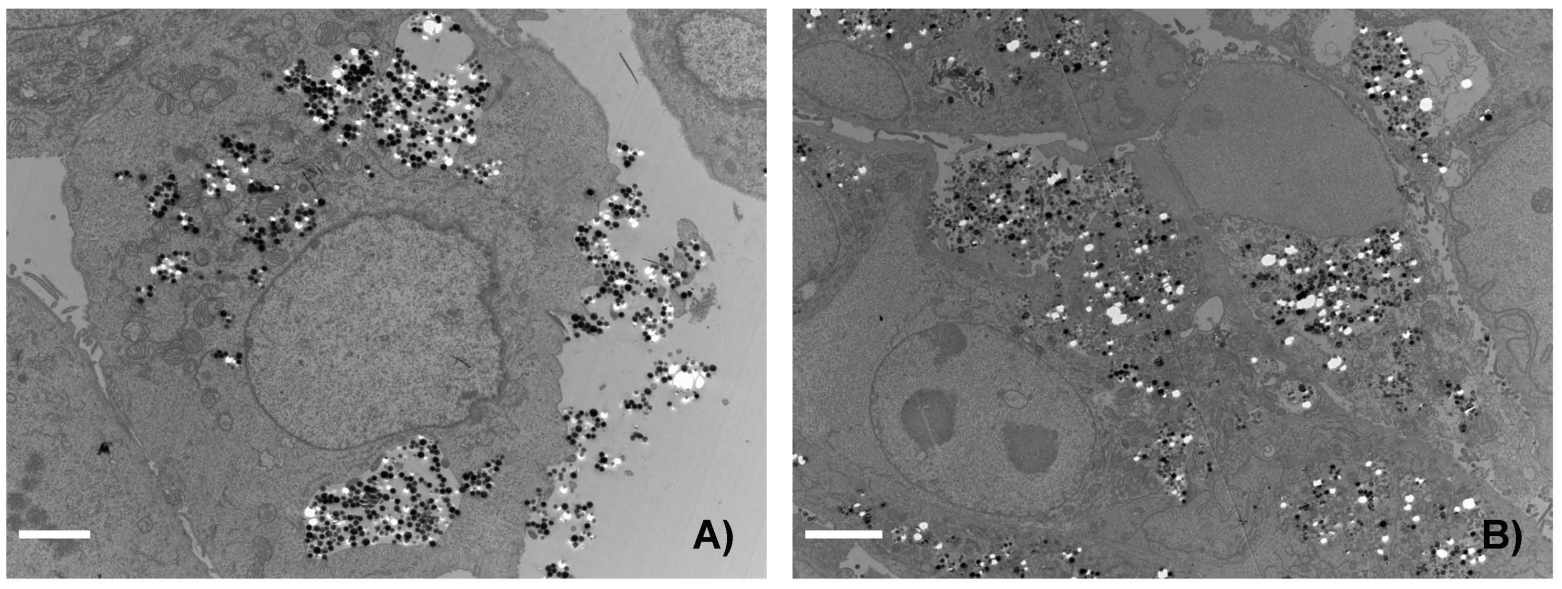
**Intracellular localisation of Ru@SiO_2_-OH and Ru@SiO_2_-NH_2 _nanoparticles after 24 h**. Transmission electron microscopy showing intracellular localisation of nanoparticles in UMB-SCC 745 after 24 h of incubation. A) Ru@SiO_2_-NH_2 _nanoparticles and B) Ru@SiO_2_-OH nanoparticles. Scale bars = 5 μm.

To obtain information about the cellular uptake of Ru@SiO_2_-PEG, Ru@SiO_2_-OH and Ru@SiO2-NH_2 _we conducted electron microscopic studies in UMB-SCC 745 cells. Generally, nanoparticle incubation did not result in an obvious ultrastructural damage compared to untreated controls. Both Ru@SiO_2_-OH and Ru@SiO_2_-NH_2 _were detected intracellularly already 30 min after nanoparticle incubation. In case of single nanoparticles, internalization involved invaginations of the plasma membrane that are lined by electron dense material at the cytoplasmic side. Furthermore, clusters of nanoparticles were internalized by membrane ruffling (Figure 2). In all cases, nanoparticles were found in membrane-bounded vesicles within the cytoplasm. Intracellular amounts of Ru@SiO_2_-OH and Ru@SiO_2_-NH_2 _nanoparticles steadily increased between 30 min and 5 h post incubation (Figure 3 and 4). However after 24 h, large vesicles with many nanoparticles were found in favour of vesicles with single nanoparticles (Figure 5). Despite multiple washing steps during sample preparation for TEM, considerable amounts of nanoparticles were attached to the cell surface at all time points investigated.

**Figure 6:**
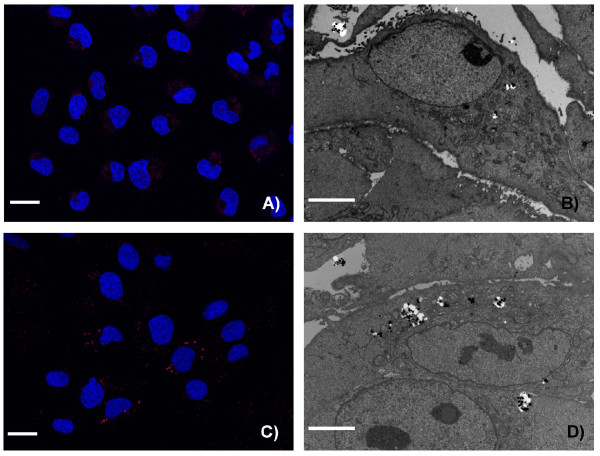
**Time dependent uptake of Ru@SiO_2_-PEG nanoparticles**. Ru@SiO_2_-PEG nanoparticle uptake after 2 h (A and B) and 24 h (C and D) in UMB-SCC 745. A, C: confocal laser scanning microscopy, showing nuclei in blue and Ru@SiO_2_-PEG nanoparticles in red, scale bars = 20 μm. B, D: transmission electron microscopy, scale bars = 10 μm.

In contrast to the other studied nanoparticles, the uptake of Ru@SiO_2_-PEG into UMB-SCC 745 cells was minimal (Figure 6). Very few Ru@SiO_2_-PEG nanoparticles were observed after 5 h of incubation and then only in a minority of cells. Neither an increase in uptake over time nor an affinity to the outer cell membrane as with the other nanoparticles could be observed. These data lead us to exclude Ru@SiO_2_-PEG from further experiments.

**Figure 7:**
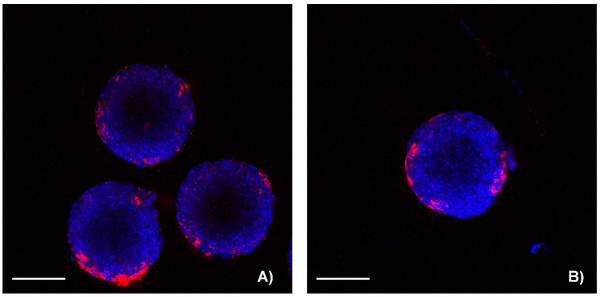
**Ru@SiO_2_-OH and Ru@SiO_2_-NH_2 _nanoparticle uptake in multicellular spheroids**. Uptake of nanoparticles in UMB-SCC 745 multicellular spheroids after 5 hours. Confocal laser scanning microscopy pictures, showing Ru@SiO_2_-OH (A) and Ru@SiO_2_-NH_2 _(B) in red and cell nuclei in blue. Scale bars = 100 μm.

The uptake of Ru@SiO_2_-OH and Ru@SiO_2_-NH_2 _nanoparticles had been additionally investigated in a 3D cell culture system (Figure 7). Confocal microscopy revealed that an intense [Ru(bpy)_3_]Cl_2 _fluorescence was visible after 5 h in the cytoplasm of cells constituting the outer layer of spheroids while inner cells were devoid of such signals.

**Figure 8:**
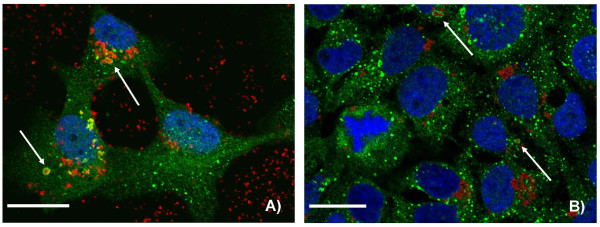
**Co-localisation of Ru@SiO_2_-OH and Ru@SiO_2_-NH_2 _nanoparticles with early endosomes**. Confocal laser scanning microscopy pictures showing a partial co-localisation after 2 h of incubation of Ru@SiO_2_-OH (A, in red) or Ru@SiO_2_-NH_2 _(B, in red) fluorescence with immunosignals for early endosomes protein 1 (A, B, in green). Cell nuclei are stained in blue. Arrows denote large early endosomes, which contain high amounts of nanoparticles. Scale bars = 30 μm.

With the aim to better characterize the intracellular fate of nanoparticles, immunohistochemical studies with antibodies against markers of endocytotic pathways were performed. CLSM analyses showed that at all time points investigated immunoreactions for Rab7, GP 120, Mitotracker and Lysotracker were present, but never co-localized with Ru@SiO_2_-OH or Ru@SiO_2_-NH_2 _nanoparticles. In contrast, a subfraction of EEA1 immunosignals coexisted with Ru@SiO_2_-OH and Ru@SiO_2_-NH_2 _fluorescence after 2 h of incubation, reaching a maximum at 5 h (Figure 8). This observation was slightly more pronounced in Ru@SiO_2_-OH. However, the majority of [Ru(bpy)_3_]Cl_2 _fluorescent nanoparticles was not located together with EEA1 immunoreactivity. Co-localization with EEA1 after 24 h of incubation was negligibly low for both nanoparticle types, even if it was slightly higher for Ru@SiO_2_-OH.

**Figure 9:**
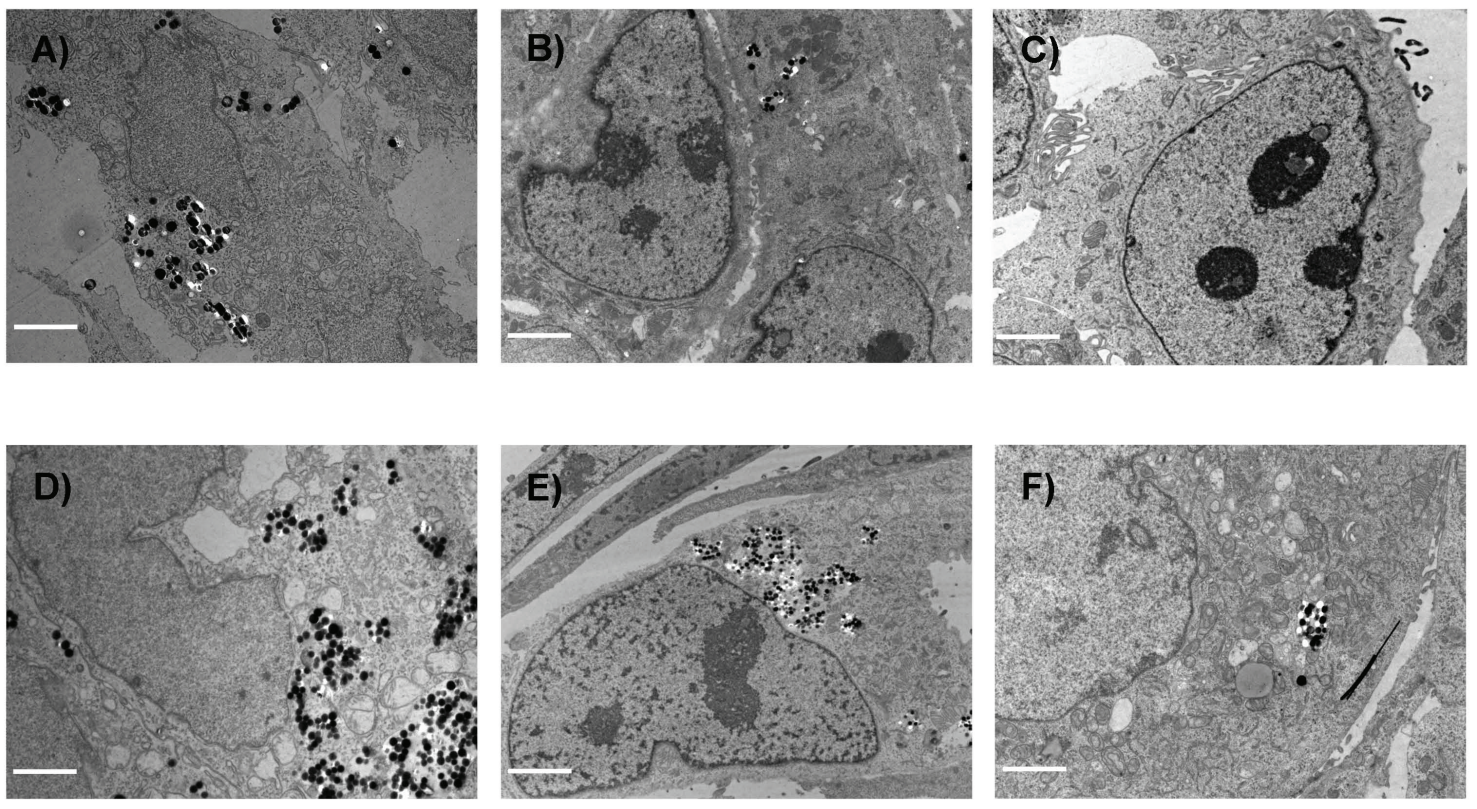
**Intracellular long time retention of nanoparticles**. Transmission electron microscopy pictures of UMB-SCC 745 after nanoparticle incubation over a time period of 15 days. A-C) 2, 9 and 12 days after incubation of Ru@SiO_2_-OH nanoparticles. D-F) 2, 9 and 12 days after incubation of Ru@SiO_2_-NH_2 _nanoparticles. Scale bars for A-E) = 5 μm and for F) = 0.5 μm.

**Figure 10:**
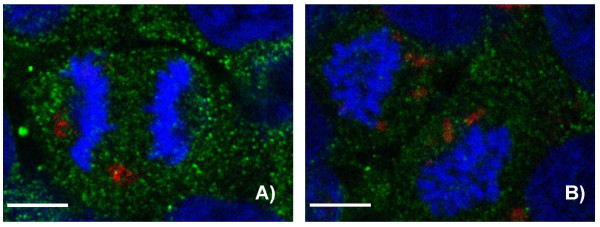
**Nanoparticle distribution during cell division**. Confocal laser scanning microscopy showing distribution of RuSiO_2_-NH_2 _nanoparticles during cell division (third passage) of UMB-SCC 745. A) metaphase and B) telophase, nucleus in blue and nanoparticles in red. Scale bars = 10 μm.

In addition, we investigated the presence of Ru@SiO_2_-OH or Ru@SiO_2_-NH_2 _nanoparticles over a time span of 15 days (i.e. over five cell passages) in UMB-SCC 745 cells (Figure 9). During the whole experiment no signs of degradation of Ru@SiO_2 _nanoparticles could be observed. During the first two days after Ru@SiO_2_-OH or Ru@SiO_2_-NH_2 _incubation all cells contained large numbers of nanoparticles. However, at day four, Ru@SiO_2_-OH nanoparticles were detected only in about 50% of cells, while Ru@SiO_2_-NH_2 _nanoparticles were still present in more than 70% of the cell population. Nine days after incubation, Ru@SiO_2_-OH and Ru@SiO_2_-NH_2 _nanoparticles were visible in less then 30% and about 50% of all cells, respectively. Generally, we found that during mitosis nanoparticles were either only propagated to one daughter cell or distributed between both daughter cells (Figure 10). At day 12 all cells exhibited a cytoplasm free of Ru@SiO_2_-OH. In contrast, Ru@SiO_2_-NH_2 _nanoparticles were found up to day 15, however, the detectable amounts were low.

## 4. Discussion

The large data corpus of recent years provides evidence that silica nanomaterials may have the potential to strongly improve cancer treatment and diagnosis. Silica nanomaterials feature the versatility necessary for tumor-specific modifications, stability in the often harsh environments of the body, ease of production and - more importantly - they are generally regarded as biocompatible. However, the latter clearly depends on many parameters such as particle size, surface modification, dose, exposure time or cell type used as model [33]. With the aim to explore the suitability of silica nanoparticles for new concepts in the treatment of head and neck cancers we investigated as a first step the biological in-vitro behaviour of non-targeted 200-300 nm core-shell silica nanoparticles with three different surface modifications.

While both Ru@SiO_2_-OH and Ru@SiO_2_-NH_2 _nanoparticles displayed high uptake rates in our model, internalization of PEGylated silica nanoparticles was almost completely lacking under the same experimental conditions. Although we observed this effect in the related HNSCC line UMB-SCC 969 and in the human prostate carcinoma cell line PC-3 as well (unpublished data), other studies showed, in contrast to our results, that PEGylated silica nanoshells are at least able to attach to the outside of MCF-7 cells [34]. However, PEG is known for its cell-repelling properties [35-37], but uptake efficiency may be increased by the addition of targeting ligands [38]. Since grafting of nanoparticles with PEG has been reported to be advantageous for in-vivo applications - basically due to its increased half-live in circulation - and helpful for targeting, the generation of optimized Ru@SiO_2_-PEG may be worthwhile (work in preparation).

Although the plasma membrane is negatively charged, the different surface charges of (negatively charged) Ru@SiO_2_-OH and (positively charged) Ru@SiO_2_-NH_2 _nanoparticles had no considerable influence on cellular uptake kinetics in our model. This is in contrast to reports indicating that negatively charged nanomaterials are less effectively internalized [39]. However, a large number of studies show that both cationic and anionic nanoparticles are capable of effectively passing the cell membrane [39].

Our data indicates that at nanoparticle concentrations of 0.125 mg/ml and below, no perturbances in cell cycle progression have been detected under our experimental conditions. An increase of cancer cell proliferation could be dangerous and hold dire consequences in clinical settings. This phenomenon has been reported in-vitro for melanoma cells and mesoporous silica nanoparticles [40], but has never been observed in our experiments. However, higher concentrations of Ru@SiO_2_-OH and Ru@SiO_2_-NH_2 _lead to reduced proliferation rates. While a slowdown in growth of tumor cells may be generally regarded as a positive effect in cancer treatment it should be emphasized that the underlying pathomechanisms in HNSCC are not clear yet. Previous in-vitro studies in other cancer cell lines have shown that cytotoxicity of silica nanoparticles, in relation to size and incubation time, may be due to oxidative stress with lipid peroxidation and membrane damage and/or an inflammatory response [41,42]. A detailed analysis of the complex molecular pathways involved is therefore needed in order to estimate possible (wanted or unwanted) consequences for future therapeutic strategies. Because of the different experimental design (e.g. longer incubation times, different particle sizes, other cell lines) it is impossible to directly compare our cytotoxicity data with previous studies. However, head and neck cancer cells seem to display cell toxic effects at concentrations comparable to other cancer cells, e.g. cervical adenocarcinoma cells [43], osteosarcoma cells [42], lung adenocarcinoma cells [37,41], and gastric and colon cancer cells [44]. Despite this, nanoparticle concentrations have to be carefully adjusted: using the same nanoparticles and experimental conditions as here, PC-3 human prostate cancer cells displayed a proliferation stagnation of about 15 days after nanoparticle incubation, although metabolic rates have been found to be higher (Besic Gyenge et al., unpublished).

With regard to internalization processes of nanoparticles into cells, phagocytosis, pinocytosis and caveolin- or clathrin-driven endocytosis have all been proposed and seem to strongly depend on particle form, size and cell type used. With our experimental set-up, apparently two different routes of nanoparticle uptake occur in parallel: on the one hand, single particles enter HNSCC cells via membrane invaginations that ultrastructurally resemble clathrin-coated pits. The involvement of clathrin-coated pits in internalization mechanisms of silica nanoparticles had also been proposed in several previous in-vitro studies using specific inhibitors or confocal methods [45-48]. On the other hand, the observed bulk internalization of nanoparticles is likely related to non-clathrin mediated endocytosis. The latter process rather displays features of macropinocytosis, such as membrane ruffling. Notably, the different surface charges of our nanoparticles did not play an apparent role with regard to the observed uptake mechanisms. Detailed studies are now needed to further characterize the events taking place at the plasma membrane upon contact with our silica nanoparticles. However, the incidence of such different simultaneous endocytosis modes of silica nanoparticles is in accordance with a recent paper, where also discrete entry pathways have been observed for single and agglomerated amorphous silica nanoparticles [48]. Furthermore, in mouse melanoma cells, internalization of latex particles of 200 nm (that corresponds approx. to the size of our particles) involved clathrin-coated pits, while latex particles of 500 nm (that corresponds approx. to our nanoparticle clusters) preferentially entered the cells via a clathrin-independent caveolin-associated pathway [49].

To characterize the intracellular fate of our silica nanoparticles within HNSCC, we next investigated their possible delivery into early and late endosomes and lysosomes. The localization of Ru@SiO_2_-OH and Ru@SiO_2_-NH_2 _in early endosomes indicates their processing to endocytotic pathways, however, a considerable number of particles obviously used a different route of trafficking, that did not involve EEA1-positive organelles. As long as these organelles have not been characterized, a possible role of nanoparticle's surface charge for endocytic processes cannot be defined. However, the acidic pH of early endosomes may explain the slightly higher frequency of (negatively charged) Ru@SiO_2_-OH in EEA1-containing vesicles.

While we cannot exclude that some Ru@SiO_2_-OH and Ru@SiO_2_-NH_2 _may have been shuttled back to the plasma membrane for segregation, the majority of nanoparticles remained intracellularly and accumulated in rather large vesicles 24 h after incubation. We propose that the latter is related to homotypic vesicle fusion. No transfer to Golgi apparatus-related pathways has been detected. More importantly, we found that nanoparticle-bearing vesicles did neither mature from early endosomes into (Rab7-positive) late endosomes nor locate to lysosomes. While both the known stability of silica-shell nanoparticles and possible cancer-related changes in endosomal sorting mechanisms may have prevented their targeting to degradation pathways, our data is in contrast to other studies showing that silica nanoparticles are in fact transferred to lysosomes [46,47,50]. Our results also differ from those of Rejman et al. [49] where a size-dependency of endocytotic pathways had been proposed. In this study, at least smaller latex particles (200 nm) passaged to late endosomes/lysosomes while only large particles (500 nm) did not [49]. We therefore conclude that intracellular fate of nanoparticles not only depends on their size (or agglomeration status) but presumably also on cell line.

Although the exact nature of different endocytotic organelles in our model has to await further characterization, the strictly vesicle-associated occurrence of Ru@SiO_2_-OH and Ru@SiO_2_-NH_2 _in HNSCC may have contributed to their biocompatibility. In human melanoma cells it had been reported that an escape of silica nanoparticles to the cytoplasm resulted in changes of the cytoskeleton as well as of adhesion and migration properties [51]. Whether the vesicular enclosure of our nanoparticles is a useful feature in the case of intracellular drug delivery strategies in HNSCC remains to be proven.

In addition to their relatively large diameter [52,53], the absence of free Ru@SiO_2_-OH and Ru@SiO_2_-NH_2 _within the cytoplasm of HNSCC may have been the reason that nanoparticles never passed the nuclear membrane. Even though localizations of silica nanoparticles within the nucleus had been observed before [54], our data is in accordance with results from previous studies [53,55,56]. Recently, it had been shown that labeling with fluorophores may affect uptake kinetics and intracellular pathways of certain probes [57]. However, due to encapsulation of the dye in our study, it is unlikely that [Ru(bpy)_3_]Cl_2 _may have influenced routes of nanoparticles within cells. Until now, very little is known about the intracellular long-term fate of silica nanoparticles and possible consequences of their persistence in biological systems. In human lung epithelial cells, Stayton et. al. observed a slow but active transfer of silica nanoparticles from the cytoplasm to the exterior environment [58]. They showed that during the first 24 h almost 50% of nanoparticles exited the cells. In contrast, our data implicates that both internalized Ru@SiO_2_-OH and Ru@SiO_2_-NH_2 _remain within the cell and are apparently distributed between daughter cells at cell division. During the course of several passages, initially high nanoparticle amounts in individual cells become "diluted", but ultrastructurally are still found in vesicles. The reason for the observed differences in long-time persistence of Ru@SiO_2_-OH and Ru@SiO_2_-NH_2 _are not clear yet, but may also be related to as yet uncharacterised charge-dependent effects of nanoparticles on endolysosomal pathways. However, in addition of not featuring acute cell toxic effects, the presence of our silica nanoparticles over 15 days caused no visible changes in viability, proliferation or morphology in HNSCC. Of note, over the time course studied, ultrastructure of nanoparticles appears to remain unchanged. However, it cannot be excluded that discrete processes of nanoparticle degradation occurred. Recently, it had been reported that, depending on functionalization, integrity of silica nanoparticles may be impaired step-wise over time in simulated body fluid with regard to e.g. surface area, pore width or pore volume [59,60].

Although the high uptake efficiency of Ru@SiO_2_-OH and Ru@SiO_2_-NH_2 _in our in-vitro mono-layer model was promising, optimized conditions are needed in case of solid HNSCC tumors where conditions of poorer vascularisation may exist. Our results in HNSCC spheroids, an established minitumor model, show that penetration depth of Ru@SiO_2_-OH and Ru@SiO_2_-NH_2 _does not reach beyond the first (outer) cell layer - independent of nanoparticle surface charge. This observation provides further evidence that our nanoparticles are not actively exocytosed. Stayton et. al. showed in-vitro that nanoparticles which were exocytosed in growth medium were taken up by other cells if not removed from growth medium [58]. Given that nanoparticles are not transported transcellularly and apparently are incapable of passing the intercellular junction complexes, new delivery strategies have to be developed for multicellular poorly vascularized cancers.

## 5. Conclusion

In summary, our study is the first to provide evidence that core-shell silica nanoparticles may be useful tools for the development of novel therapeutic strategies with cancers of the head and neck region. However, before an encapsulation of pharmaceutical compounds or a functionalization with targeting and imaging moieties may be considered, a better understanding of how these nanoparticles interact with HNSCC cells on contact and after internalization is needed. Starting from our first steps towards clarification of endocytic pathways, further microscopic, immunocytochemical and molecular biological studies will elucidate nanoparticle sorting as well as their further intracellular fate, including possible degradation processes, or nanoparticle-mediated molecular cell responses.

## Competing interests

The authors declare that they have no competing interests.

## Authors' contributions

CM and EBG designed all of the experiments and wrote the manuscript. EBG and XD performed all the experiments together. HW and MB contributed clinical expertise. AWH and UP synthesized and characterized the nanoparticles. All authors read and approved the final manuscript.
